# Complex resistance of spring and winter bread wheat lines to biotic and abiotic stresses

**DOI:** 10.18699/VJ21.082

**Published:** 2021-11

**Authors:** I.F. Lapochkina, N.R. Gainullin, О.A. Baranova, N.M. Kovalenko, L.A. Marchenkova, O.V. Pavlova, O.V. O.V. Mitroshina

**Affiliations:** Federal Research Center “Nemchinovka”, Novoivanovskoe, Moscow region, Russia; Federal Research Center “Nemchinovka”, Novoivanovskoe, Moscow region, Russia; All-Russian Institute of Plant Protection, Pushkin, St. Petersburg, Russia; All-Russian Institute of Plant Protection, Pushkin, St. Petersburg, Russia; Federal Research Center “Nemchinovka”, Novoivanovskoe, Moscow region, Russia; Federal Research Center “Nemchinovka”, Novoivanovskoe, Moscow region, Russia; Federal Research Center “Nemchinovka”, Novoivanovskoe, Moscow region, Russia

**Keywords:** common wheat, stem and leaf rust, spot blotch and tan spot, salt resistance, resistance to hypoxia, мягкая пшеница, стеблевая и бурая ржавчины, темно-бурая и желтая пятнистости, солеустойчивость, устойчивость к гипоксии

## Abstract

An original initial material of spring and winter bread wheat with group resistance to stem and leaf rust was developed using new donors of resistance to stem rust: winter soft wheat GT 96/90 (Bulgaria) and accession 119/4-06rw with genetic material of the species Triticum migushovae and (Aegilops speltoides and Secale cereale), respectively, a line of spring wheat 113/00i-4 obtained using the species Ae. triuncialis and T. kiharae, as well as spring accession 145/00i with genetic material of the species Ae. speltoides resistant to leaf rust. The transfer of effective Sr- genes to progeny was monitored using molecular markers. New lines underwent a f ield assessment of resistance to leaf and stem rust in the epiphytotic development of diseases in the Central Region of the Russian Federation, as well as in the North Caucasus and Western Siberia, and showed high resistance to these pathogens. Fourteen genotypes of spring wheat with group resistance to these diseases and parental forms that participated in the origin of the lines were evaluated for resistance to spot blotch (Cochliobolus sativus) and tan spot (Pyrenophora tritici-repentis) using isolates from Kazakhstan and Omsk in laboratory conditions. A highly resistant parental form of winter soft wheat from “Arsenal” collection 119/4-06rw (wheat-Ae. speltoides-rye hybrid 2n = 42) with group resistance to two spots, four medium-resistant genotypes to both isolates of tan spot from Kazakhstan and Omsk populations of the pathogen, as well as genotypes resistant to the Omsk isolate of P. tritici-repentis (parental form 113/00i-4 and lines 1-16i, 6-16i, 9-16i) were isolated. Among the lines of winter wheat, four were identif ied with group resistance to spot blotch and tan spot. Additionally, the stress resistance of the lines to NaCl salinization and prolonged f looding of seeds with water was evaluated at the early stages of ontogenesis in laboratory conditions. Lines 33-16i, 37-16i, 32- 16i and 9-16i showed a high ability to withstand excess moisture. Lines 33-16i, 37-16i, 32-16i and 3-16i were characterized by high salt tolerance, exceeding the average of 49.7 %. Among the winter genotypes, lines were identif ied with increased resistance to hypoxia (37-19w, 32-19w, 16-19w, 90-19w) and with increased salt tolerance (20- 19w, 9-19w, 37-19w, 90-19w), signif icantly exceeding the standard cv. Moskovskaya 39. The listed lines are of interest as sources of resistance to anaerobic and salt stress, as well as donors of resistance to a group of fungal diseases: leaf and stem rust and tan spot. We attribute the increased level of resistance of the new initial material to the presence of alien translocations in the original parental forms involved in the origin of the lines.

## Abstract (ru)

Оригинальный исходный материал яровой и озимой мягкой пшеницы с групповой устойчивостью к стеблевой и бурой ржавчинам создан с использованием новых доноров устойчивости к стеблевой ржавчине – озимой мягкой пшеницы GT 96/90 (Болгария) и линии 119/4-06rw с генетическим материалом соответственно видов Triticum migushovae и (Aegilops speltoides и Secale cereale), линии яровой пшеницы 113/00i-4, полученной с привлечением видов Ae. triuncialis и T. kiharae, а также ярового образца 145/00i с генетическим материалом вида Ae. speltoides, устойчивого к бурой ржавчине. Передачу эффективных Sr-генов потомству отслеживали с помощью молекулярных маркеров. Новые линии прошли полевую оценку устойчивости к бурой и стеблевой ржавчине при эпифитотийном развитии болезней в Центральном регионе Российской Федерации, а также на Северном Кавказе и в Западной Сибири и показали высокую устойчивость к этим патогенам. Четырнадцать генотипов с групповой устойчивостью к этим болезням и родительские формы, принимавшие участие в происхождении линий, в лабораторных условиях оценили на устойчивость к темно-бурой (Cochliobolus sativus) и желтой (Pyrenophora tritici-repentis) пятнистостям c применением изолятов из Казахстана и Омска. Выделена высокоустойчивая родительская форма озимой мягкой пшеницы из коллекции «Арсенал», 119/4-06rw (пшенично-эгилопсно-ржаной гибрид 2n = 42) с групповой устойчивостью к двум пятнистостям и четыре среднеустойчивых генотипа к обоим изолятам желтой пятнистости (из казахстанской и омской популяций патогена), а также генотипы, устойчивые к омскому изоляту P. tritici-repentis (родительская форма 113/00i-4 и линии 1-16i, 6-16i, 9-16i). Среди образцов озимой пшеницы выделено четыре с групповой устойчивостью к темно-бурой и желтой пятнистостям. Дополнительно оценена стрессоустойчивость линий в лабораторных условиях на ранних этапах онтогенеза к засолению NaCl и длительному затоплению семян водой. Линии 33-16i, 37-16i, 32-16i и 9-16i проявили высокую способность противостоять избытку влаги. Высокой солеустойчивостью, превышающей средний показатель, 49.7 %, характеризовались линии 33-16i, 37-16i, 32-16i и 3-16i. Среди озимых генотипов выделены образцы с повышенной устойчивостью к гипоксии, 37-19w, 32-19w, 16-19w, 90-19w, и солеустойчивостью – 20-19w, 9-19w, 37-19w, 90-19w, достоверно превышающие стандартный сорт Московская 39. Эти образцы представляют интерес как источники устойчивости к анаэробному и солевому стрессу, а также как доноры устойчивости к группе грибных заболеваний: бурой и стеблевой ржавчинам и желтой пятнистости листьев. Повышенный уровень устойчивости нового исходного материала мы связываем с наличием чужеродных транслокаций у исходных родительских форм, участвовавших в происхождении линий.

## Introduction

The Non-Black Earth Region belongs to the zone of insecure agriculture, which has always been full of abiotic and biotic stress factors. Predominant fungal diseases are powdery mildew, leaf rust, and in 2010 stem rust, which had not been present for 27 years, returned to the fields. In recent years, due to global warming, wheat crops are periodically affected by leaf spots (spot blotch and tan spot) and septoria. The harmfulness of these diseases is high and yield losses can reach 40–50 % (Afanasenko et al., 2011; Mikhailova et al., 2012; Kim, Volkova, 2020).

On the one hand, among abiotic stresses, frequent May droughts that lead to crops getting thinned are observed, and, on the other hand, excess moisture, flooding of crops during snowmelt, snow mold damage are possible. Frequent heavy rainfall during growing season leads to lodged crop. Despite the significant success of breeders in creating highly productive varieties of spring and winter wheat for this zone, the development of the varieties resistant to biotic and abiotic environmental factors remains relevant, especially in recent decades, when we’ve been facing real facts of climate change leading to a change in the species spectrum of phytopathogenic fungi and their racial composition (Lekomtseva et al., 2007, 2008; Zeleneva et al., 2021).

The main goal of our research was the development of productive competitive spring and winter wheat lines resistant to stem rust Puccinia graminis f. sp. tritici (Pgt) and other dangerous pathogens (P. triticina, Blumeria graminis, Pyrenophora tritici-repentis, Cochliobolus sativus) and the identification of other economically valuable qualities and traits of the obtained material. The strategy and tactics of developing such an initial material were based on the previously created “Arsenal” bread wheat collection (Lapochkina, 2005), represented by genotypes with supplemented Aegilops speltoides chromosomes and alien translocations Ae. speltoides, Ae. triuncialis, Triticum kiharae and Secale cereale, as well as the search for new sources of resistance to the Ug99 stem rust race.

## Materials and methods

History of the development of wheat lines with increased resistance to rust fungi began in 2010, when part of the “Arsenal” collection (90 accessions), as well as accessions from All-Russian Research Institute of Plant Genetic Resources (VIR) (129 accessions) were evaluated at the University of Minnesota for resistance to stem rust of the Ug99 race at the seedling stage. Seven genotypes of bread wheat with 2n = 42 and 2n = 44 from the “Arsenal” collection, as well as several genotypes from the VIR collection that showed resistance to this dangerous pathogen (type of reaction to the penetration of the fungus 0;, 1, 2) were selected. For further study and hybridization hexaploid accessions of known origin and accessions with alien material were left: winter wheat- Ae. speltoides-rye line 119/4-06rw (Ae. speltoides, S. cereale), a line from Bulgaria GT 96/90 with genetic material of the species Triticum migushchovae, winter wheat variety Donskaya polukarlikovaya (Ae. squrrossa) and a spring wheat accession 113/00i-4 with the genetic material of Ae. triuncialis and T. kiharae species.

The assessment of economically valuable characteristics in the field conditions of the Moscow region against the leaf rust infectious background highlighted a high resistance to the leaf rust population (0–5 % of severity) in all accessions. Accessions 113/00i-4 (further in Tables – 113) and 119/4-06rw (further – 119) were highly resistant to powdery mildew, and accessions from VIR (cv. Donskaya polukarlikovaya) and the GT 96/90 line) (further – D/p and 96) were susceptible to this disease. However, they had other economically valuable traits: precocity (early heading) and short stem. All accessions were productive enough not to cause concerns about a decrease in productivity during hybridization.

The Sr genes were identified in the accessions using molecular markers recommended for marker-assisted selection (MAS). Molecular markers to 11 Sr genes were used: Xgwm533 – Sr2 (Hayden et al., 2004); STS638 – Sr15 (Neu et al., 2002); Wpt5343 – Sr17 (Crossa et al., 2007); Xbarc121, Xcfa2123, Xcfa2019 – Sr22 (Khan et al., 2005; Yu et al., 2010); Sr24#12, Sr24#50 – Sr24/Lr24 (Mago et al., 2005); Scm9 – Sr31 (Weng et al., 2007); Xbarc55, Xstm773 – Sr32 (Somers et al., 2004; Dundas et al., 2007; Yu et al., 2009); Xwmc477, Xstm773-2 – Sr36 (Tsilo et al., 2008); Sr39#22 – Sr39 (Mago et al., 2009); Xgwm344 – Sr40 (Wu et al., 2009); Xgwm501 – Sr47 (Faris et al., 2008).

The PCR terms are given in the original works, but optimal conditions for each marker were selected. Both effective and non-effective genes for the Ug99 stem rust race, but showing resistance in the Non-Black Earth Region and the North Caucasus were identified (Baranova et al., 2015). Since we had three winter genotypes and only one spring genotype at our disposal, we considered the strategy for developing hybrid populations with a spring and winter pattern of life using different plant growing backgrounds

Initially, the parental forms were crossed taking into account their alternative characteristics. Namely: a tall source was crossed with a short-stemmed one; a late-maturing source – with an early-earing one; a genotype resistant to powdery mildew was crossed with a susceptible one. In the first year, direct and reverse crosses of three samples were performed (GT 96/90, 119/4-06rw and 113/00i-4). The Donskaya polukarlikovaya variety’s early start of heading made it impossible to carry out hybridization with it. F2 seeds were divided in half and grown on different backgrounds.

To obtain spring genotypes, sowing was carried out in spring in the field, and the plants that completed the heading process were pollinated either with a recurrent parental form – line 113/00i-4 or with accession 145/05i, which was resistant to powdery mildew and leaf rust, but susceptible to stem rust.

The second half of the seeds was sown in heated ground in February. After seedling emergence, heating was turned off. The plants were vernalized and went through heading in natural conditions. Then, depending on their habitus, they were pollinated either with the Donskaya polukarlikovaya variety, with the GT96/90 line or the 119/4-06rw winter line. The use of heated background, conventional sowing and sowing in greenhouse vessels allowed to speed up the process of obtaining back-cross progeny of various saturation levels.

After self-pollination, individual plants with traits of resistance to leaf rust and powdery mildew, as well as with other valuable traits, were selected from this progeny against the infectious background of leaf rust. Identification of stem rust resistance genes of these plants was conducted using the molecular markers listed above, and plants with several resistance genes in a homozygous state and a complex of economically valuable characteristics were selected for field tests of resistance in the Moscow and Krasnodar Krai regions – as well as in Western Siberia (Omsk).

Immunological assessment of the lines resistance to stem rust in the Central and West Siberian regions was carried out to the natural population of the fungus in field conditions, and in the Krasnodar Krai region – against an artificial infectious background of stem and leaf rust development. North Caucasian populations of Puccinia spp were used as infectious material in the latter case. Plant damage level was recorded during the period of maximum development of diseases. The evaluation criteria were the type of reaction and the plant damage level according to the scale recommended by CIMMYT (Roelfs, Singh, 1992).

The resistance to spot blotch (Cochliobolus sativus) and tan spot (P. tritici-repentis) of the parental forms of crossing and lines with a complex of economically valuable traits was determined. For the latter, two isolates selected from P. tritici-repentis populations common in Western Siberia were used: (Omsk isolate) from the temperate climatic zone with a continental climate of forest-steppe and (Kazakhstan isolate) from the sharply continental zone of Northern Kazakhstan. Isolates differ in virulence. The assessment was carried out in laboratory conditions on leaf sections placed in the benzimidazole solution (0.004 %) according to the method of L.A. Mikhailova and co-authors (Mikhailova et al., 2012).

The stress resistance of spring and winter wheat lines to abiotic stresses, namely, water flooding (hypoxia) and NaCl salinization, was evaluated in laboratory conditions at the early stages of ontogenesis according to generally accepted methods (Beletskaya, 1976; Semushkina et al., 1976). The experiments were carried out in two replicates.

Statistical indicators and the reliability of their differences were determined in comparison with standard varieties using statistical analysis (Martynov, 1999).

## Results and discussion

As a result of Sr genes identification using molecular markers recommended for MAS, both effective and non-effective with regard to Ug99 but demonstrating resistance in the Non-Black Earth Region, genes were identified (Baranova et al., 2015) (Table 1).

**Table 1.**
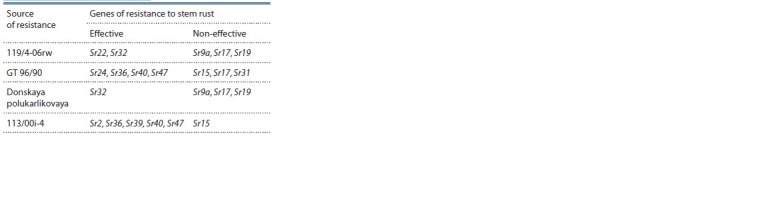
Sr genes identif ied in the sources of resistance to stem rust race Ug99

From 2 to 4 effective resistance genes were recorded in backcross progeny obtained after self-pollination. The genetic diversity of Sr genes among spring and winter wheat plants differed. Nine main gene combinations in spring plants were identified: Sr2+Sr36; Sr2+Sr39; Sr2+Sr32; Sr2+Sr22; Sr2+Sr36+Sr40; Sr2+Sr32+Sr40; Sr2+Sr22+Sr40; Sr2+Sr32+Sr39; Sr2+Sr22+Sr32+Sr40. It is twice as high in winter crops, but the frequency of occurrence of the Sr2 resistance gene of adult plants was noted only in 35 % of individual plants selected for gene identification. And in half of the cases, the Sr2 gene was in a heterozygous state. Winter wheat plants were characterized by a unique combination of resistance genes that are rarely used in the breeding process: Sr22+Sr32; Sr22+Sr47; Sr32+Sr47; Sr2+Sr22+Sr32; Sr22+Sr32+Sr40; Sr36+Sr39+Sr47. A plant with four resistance genes was revealed: Sr2+Sr22+Sr32+Sr40.

The evaluation of the progeny of 198 bread wheat spring lines and 367 lines of winter wheat with two-three Sr resistance genes was conducted in various geographical points of the Russian Federation to leaf and stem rust, differing in the spectrum of virulence genes (Lapochkina et al., 2016, 2018). This resulted in the selection of lines with group resistance to both pathogens (Table 2). Among the spring wheat lines, a high frequency of resistant genotypes to the North Caucasian population of stem and leaf rust was noted (81–82 %).

**Table 2.**
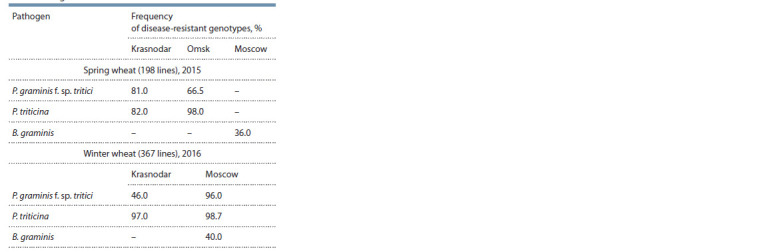
The results of the assessment of lines of spring and winter wheat to rust fungi and powdery mildew in various regions of the Russian Federation

The frequency of occurrence of resistant genotypes to the West Siberian population of stem rust was lower (66.5 %). These data were facilitated by late sowing of spring crops, which was intentionally used as a factor stimulating affection by the pathogen. Due to the drought in 2015, 167 of the 198 lines survived, 111 of them were resistant to stem rust, and almost all of the material was resistant to leaf rust. It should also be noted that Western Siberia is characterized by the presence of aggressive population of the stem rust pathogen. This was demonstrated by the assessment results of the collection of isogenic lines and cultivars with known genes of resistance to stem rust, which showed differentiation only at the first assessment of the damage, and later the results were negated due to a strong disease development (Lapochkina et al., 2016), as well as by the results of the study on the racial composition of Western Siberian populations of this pathogen (Skolotneva et al., 2020).

In the Moscow region in 2015, the development of rust fungi was not observed, and an attempt to create an artificial leaf rust background failed due to high temperatures and low humidity of air and soil. However, 71 lines (36 %) with resistance to powdery mildew have been selected this year.

The resistance of 367 winter wheat lines was evaluated under the conditions of epiphytoty development of stem rust in Krasnodar and Moscow in 2016. In Krasnodar, 168 lines resistant to P. graminis were selected; almost all lines were resistant to leaf rust. Under the conditions of stem rust epiphytotic, a high yield of resistant genotypes 96–98 % to both pathogens) was also noted in Moscow. The frequency of occurrence of genotypes resistant to powdery mildew was about 40 %.

According to the assessment results, about 70 lines of spring wheat and more than 100 winter lines combining group resistance to rust fungi with a complex of other economically valuable characteristics (early heading period, optimal height, ear productivity of 1.7–2.5 g, large grain and high protein and gluten content in the grain) were selected.

Among the lines with a complex of economically valuable characteristics, an additional assessment of resistance to pathogens that cause spot blotch and tan spots development on the leaves was carried out. In total, 14 lines of spring wheat, 9 lines of winter wheat and the initial parental forms of crossing were evaluated (Table 3).

**Table 3.**
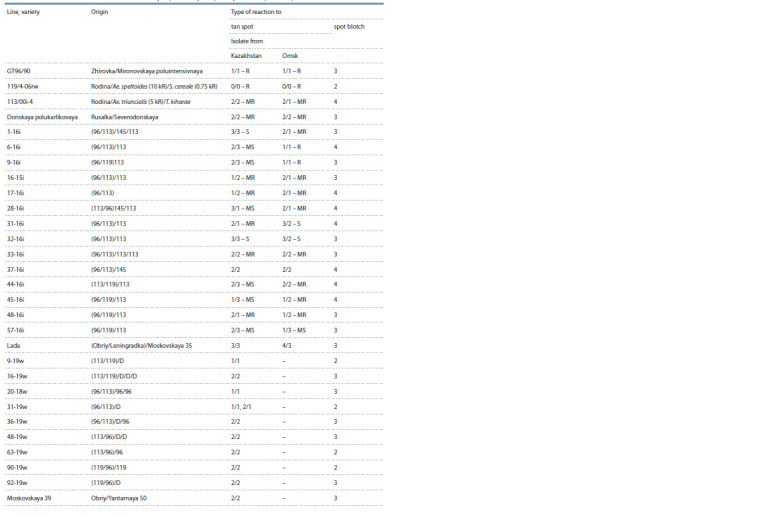
The assessment results of the sources of resistance to stem rust and lines of spring and winter wheat obtained with the involvement of the sources to tan spot (P. tritici-repentis) and spot blotch (C. sativus)

According to the evaluation results, the only mediumresistant accession 119/4-06rw from the “Arsenal” collection with reaction type 2 to spot blotch was identified. With regard to spot blotch, this is an excellent result. It is extremely rare for accessions resistant to spot blotch to be selected. Generally, reaction type 3–4 is traced in bread wheat accessions. The resistance to this pathogen is usually associated with the species T. macha, T. vavilovii, T. timopheevii, T. monococcum and T. spelta (Mikhailova et al., 2012).

Two isolates were used to infect the leaf segments with P. tritici-repentis: from Kazakhstan and Omsk. Accessions 119/4-06rw and GT 96/90 demonstrated high resistance to both isolates. High resistance to the Omsk tan spot isolate was found in the spring wheat line 113/00i-4 with the genetic material Ae. triuncialis and T. kiharae.

Among the 14 tested lines of spring wheat, no resistant genotypes to spot blotch were detected. Ten lines were resistant to the Omsk tan spot isolate, and 4 lines showed resistance to both the Kazakhstan and Omsk tan spot isolate (16i-16i, 17i-16i, 33-16i, 48-16i).

Among the 9 lines of winter wheat that got to check plant breeding nursery and competitive variety test, 4 were found to have resistance to spot blotch: 9-19w, 31-19w, 63-19w, 90-19w. Being infected with the most virulent tan spot isolate from Kazakhstan, the same four lines were selected with high resistance to P. tritici-repentis.

The stress resistance of spring and winter wheat lines to water flooding (hypoxia) and NaCl salinization was evaluated under laboratory conditions at the early stages of ontogenesis. Progeny of 11 lines of spring wheat from the Nursery that had been tested for two years and two standard varieties (the modern variety Zlata and the previous variety Lada) were tested (Table 4). A high level of variation of the resistance to hypoxia basis (CV > 40 %) was recorded. Four lines (33-16i, 37-16i, 32-16i and 9-16i) reliably exceeded the indicators of the Zlata variety with regard to the mentioned stress factor; however, like this variety, they lagged behind the breeding masterpiece of E.D. Nettevich – the Lada variety (Nettevich et al., 1996).

**Table 4.**
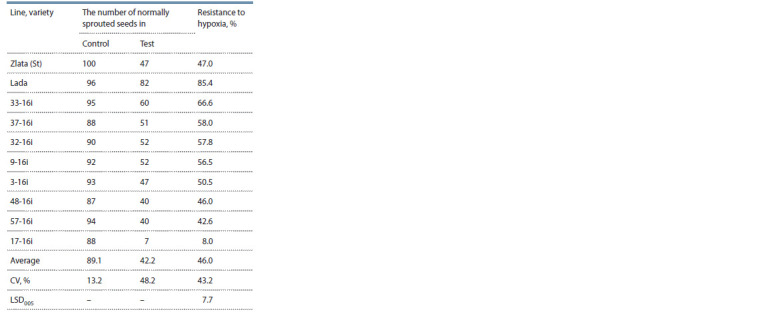
The reaction of spring wheat lines to water f looding of seeds

As for the resistance to hypoxia, about two dozen lines of winter wheat were evaluated from the check plant nursery of 2019 (the conditions for seed formation were favourable) and from Competitive Plant Nursery of 2020 (the grain was formed in conditions of heavy rainfall and lodged crops). A high level of property variation was noted: the number of normally sprouted seeds (CV > 30 %). Four genotypes with resistance above the average as of the test and standard were identified: 37-19w, 32-19w, 16-19w and 9-19w (Table 5).

**Table 5.**
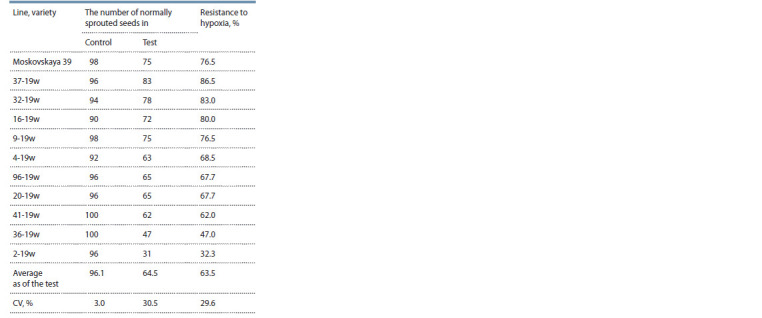
The reaction of winter wheat lines to water f looding of seeds

The results obtained with seeds of winter lines formed under unfavourable conditions in 2020 were markedly different. Only 50 % of seeds of the standard variety sprouted normally after flooding. Against this background, two lines, 90-19w and 16-19w, reliably exceeded the resistance to hypoxia level of the standard variety.

The harmful effect of NaCl salinization caused the depression of the length of seedlings in spring wheat. Both standard varieties and line 37-16i had high resistance to sodium chloride, exceeding the average results of the test (49.6 %). (Table 6). The comparison of the effect of anaerobic stress and salinization on spring wheat showed that the range of variability for salt tolerance fluctuated from 40 to 62 %, and for anaerobic stress – from 8 to 85 % that is a stronger differentiation of spring wheat genotypes with regard to water stress.

**Table 6.**
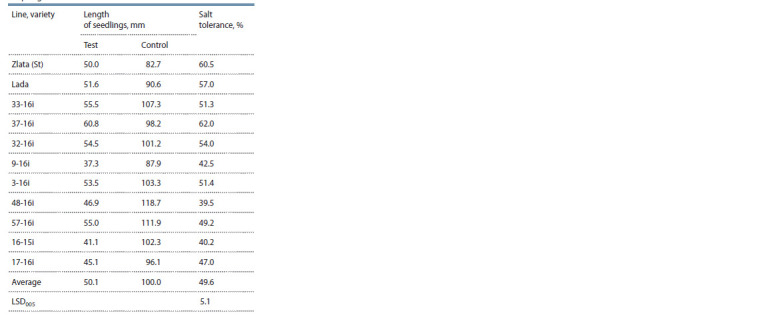
Depressive effect of NaCl on growth processes in spring wheat lines

A high level of growth depression during salinization was also noted in winter wheat lines (CV = 28 %). A high ability to resist salt stress exceeding both the average of the test and the St variety level was revealed in the lines: 20-19w, 9-19w and 37-19w (Table 7).

**Table 7.**
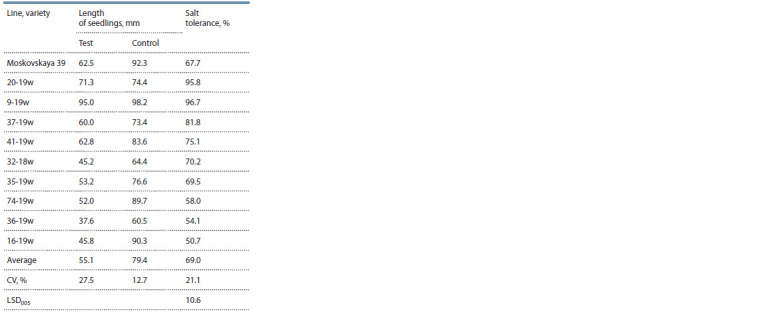
Depressive effect of NaCl on growth processes in winter wheat lines

The test with planted seeds formed in the unfavourable year of 2020 showed, on the one hand, a decrease in salt tolerance level in Moskovskaya 39 from 68 to 49 %, and, on the other hand, revealed another 90-19w line resistant to this stress, which has been in the nursery of competitive variety testing since 2021.

Some of the lines are already under additional environmental testing at the Federal Scientific Agroengineering Center VIM in the Ryazan region. Expansion of environmental testing of winter wheat lines to Western Siberia (the backcrossed progeny of individual plants was transferred to the Omsk State Agrarian University), makes it possible to develop a new winter wheat crop for this region. There is a high probability of selecting winter-hardy winter wheat lines with group resistance to fungal diseases that demonstrated a good overwintering level according to the results of assessments performed in May 2021 (see the Figure). These lines are highly likely to be resistant to rust fungi, since this material has already been evaluated for resistance to these pathogens in the Krasnodar Krai region and in the Moscow region in 2016 during the epiphytotic stem rust. Since there are genotypes that are resistant to salinization among them, there is a high probability of selecting genotypes that are resistant to drought, which is important for Western Siberia, since both resistances are correlated.

**Fig.**
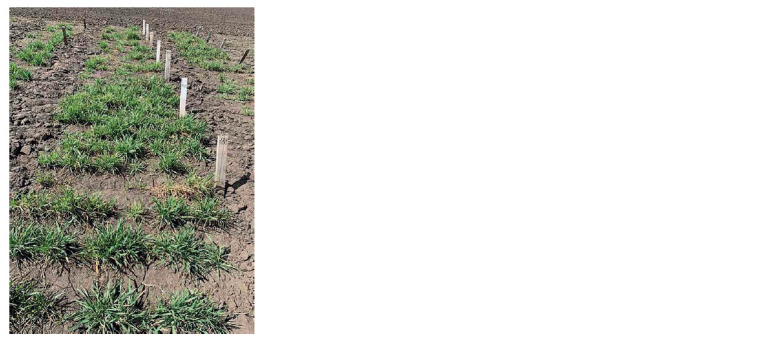
The winter wheat lines regrowth in Western Siberia in May 2021. The photo was kindly provided for publication by Professor V.P. Shamanin, Omsk State Agrarian University.

## Conclusion

After a comprehensive assessment of the obtained initial material and the identification of additional positive properties has facilitated the selection of breeding lines for reproduction and competitive variety testing. While in previous years we focused our attention on lines with a high protein and gluten content in grain and resistant to rust diseases, now we have lines with complex resistance to phytopathogens and abiotic stresses: spring wheat lines, 9-16i, 32-16i, 37-16i and 48-16i; winter wheat lines, 20-19w, 9-19w, 9-19w, as well as lines 31-19w and 48-19w with extended group resistance to fungal diseases (stem and leaf rust, powdery mildew, tan spot and spot blotch). This material can be used as a source of resistance to unfavourable environmental factors at the next stage of improving bread wheat, as well as to identify its possibility to compete for productivity with modern wheat varieties.

The obtained initial material is of interest for molecular genetic mapping of resistance and QTL genes, as well as for MAS for Sr genes, especially for genes that are rarely used to increase immunity to stem rust: Sr32, Sr39, Sr40, and Sr47.

## Conflict of interest

The authors declare no conflict of interest.
